# The Sleepiness–Depression Link in Obstructive Sleep Apnea: Preliminary Results on the Mediation of Impulsivity

**DOI:** 10.3390/jcm12206467

**Published:** 2023-10-11

**Authors:** Mariacarolina Vacca, Matteo Spanetta, Andrea Ballesio, Mariana Fernandes, Fabio Placidi, Francesca Izzi, Caterina Lombardo, Nicola Biagio Mercuri, Giuseppina Laganà, Claudio Liguori

**Affiliations:** 1 Department of Psychology, Sapienza University of Rome, 00185 Rome, Italy; mariacarolina.vacca@uniroma1.it (M.V.); andrea.ballesio@uniroma1.it (A.B.); caterina.lombardo@uniroma1.it (C.L.); 2 Santa M. Della Stella Hospital, 05018 Orvieto, Italy; spanettamatteo95@gmail.com; 3 Department of Systems Medicine, University of Rome “Tor Vergata”, 00133 Rome, Italy; mariana.fernandes@uniroma1.it (M.F.); fbplacidi@gmail.com (F.P.); mercurin@med.uniroma2.it (N.B.M.); 4 Sleep Medicine Center, Neurology Unit, University Hospital of Rome “Tor Vergata”, 00133 Rome, Italy; 5 Department of Orthodontics, University of Rome “Tor Vergata”, 00133 Rome, Italy; giuseppinalagana@libero.it

**Keywords:** OSAS, depression, impulsivity, mediation, psychopathology

## Abstract

Background: Emotional impulsivity has been found to be relevant in explaining the association between sleep problems and depressive symptoms, suggesting the potential role of impulsivity as a key underlying mechanism of this link. The objective of this study was to take a preliminary step in understanding the mediating role of impulsivity in the relation between excessive daytime sleepiness (EDS) and depression in patients with obstructive sleep apnea syndrome (OSAS) and to compare psychological and demographic characteristics between different levels of daytime sleepiness. Methods: A total of 138 patients with OSAS underwent polygraphic cardiorespiratory monitoring and completed a series of questionnaires investigating perceived sleepiness, depression, impulsivity, and other psychological characteristics. A mediational model was tested in order to assess whether impulsivity mediated the relation between sleepiness and depressive symptoms while controlling for the effects of age, sex, BMI, and oxygen saturation parameters. Results: the mediation model showed that there was a significant indirect effect of impulsivity in the sleepiness–depression link (αβ = 0.084 [0.0243–0.1617]). Conclusions: The here-presented results showed that the sleepiness–depression link is not direct as previous studies asserted, but instead it may be better explained by impulsivity. Research and practical implications are discussed.

## 1. Introduction

The International Classification of Sleep Disorders (ICSD-3) defines obstructive sleep apnea syndrome (OSAS) as sleep-disordered breathing (SDB) characterized by (1) an apnea–hypopnea index (AHI) ≥ 5 h and ≥one OSA symptom or associated medical/psychiatric disorder; (2) an apnea–hypopnea index (AHI) ≥ 15 h [1]. Patients with OSAS present a repetitive complete or partial obstruction of the upper airway during sleep, resulting in sleep fragmentation and oxygen desaturation [2]. The gold-standard diagnostic tool for diagnosis of OSAS is the laboratory polysomnography (PSG), as stated by the Standards of Practice Committee of the American Sleep Disorders Association [3]. According to several population-based studies, the prevalence of OSAS diagnosis is relatively considerable [4].

A recent systematic review conducted on 24 prevalence studies (i.e., 14 European and 5 North American studies) indicated an occurrence of OSA ranging from 9% to 38% based on AHI [5]. In particular, evidence on OSA prevalence in Italy revealed an alarming percentage, with moderate-severe conditions (AHI ≥ 15 h) affecting between 9% and 27% of the population (e.g., 4 to 12 million patients) aged 15–74 years [6]. The attention towards OSA is increasing [6] not only for its high prevalence rates but also considering its association with decreased quality of life [6].

For example, a large proportion of patients with OSAS (16 to 55%) often report depressive symptoms [2]. Depression is a mood disturbance involving negative cognitive and emotional functioning; it is mostly characterized by a daily persistent sad mood accompanied by a loss of interest in regular pleasurable activities [7].

Several cross-sectional studies evidenced the co-occurrence between OSA and clinically significant depression, e.g., [8], and longitudinal research has demonstrated a prospective link between OSA severity and the subsequent risk of developing depression, e.g., [9].

Previous research has analyzed the association between clinical and personal characteristics presented by patients with OSAS and the higher likelihood of reporting depressive symptoms, claiming that some OSA cases may represent a related risk factor for depression. For example, depression has been found to be more prevalent in female than in male individuals with OSA [10,11], suggesting an association between sex and the occurrence of depression in these patients. Moreover, high body mass index (BMI) [12] and excessive daytime sleepiness (EDS) [13] have been implicated in the greater manifestation of depression in OSA [4]. Some authors argued that the frequently observed OSA comorbidity with depression could involve the link between some mechanisms presented in OSA, such as cardiovascular diseases or metabolic imbalance, and elevated levels of individual mental distress [4].

Even though depressive symptoms are unquestionably involved in the overall expression of OSA, the processes underlying the association between OSAS and depression have yet to be fully investigated [14]. Possible mechanisms may involve daytime sleepiness (i.e., a key symptom in OSAS) [15] and impulsivity (i.e., a tendency to engage in non-reflective reactions to internal or external stimuli) [16].

Daytime sleepiness can intrude into daily activities in OSA, affecting individual affection, cognition, and quality of life [17]. It has been reported that 40.5–58% of patients with OSAS report EDS at initial diagnosis [18], although a notable amount (9–22%) were found to present residual EDS even when OSA treatment with continuous positive airway pressure (CPAP) therapy was started [19]. A large number of patients with depression have reported concomitant daytime sleepiness [20], and some authors highlighted the strong relationship between daytime sleepiness and suicidal ideation [21]. EDS, especially when persistent, can prospectively predict high depressive symptoms [22], suggesting the urge of clinicians to evaluate the underlying process explaining this relationship that, to date, remains uncertain. In this regard, impulsivity could be a potential mediator candidate. Impulsivity has been defined as the predisposition to react without forethought despite the negative consequences [5]. Previous evidence has revealed that impulsivity positively correlates with individual mental distress, particularly with depressive symptoms [23] and sleep disturbances [24].

It has been suggested that the hypoxia and sleep difficulties associated with OSA can lead to higher impulsivity as a result of prefrontal cortical dysfunction [24], suggesting that impulsivity may play a key role in understanding psychological functioning in OSA patients. High impulsivity has previously been associated with high sleep deprivation, as the ability to inhibit an undesirable response depends on the integration of several frontal loci that are vulnerable to sleep loss [23]. Some of the typical symptoms reported by OSA patients could be involved in the enhancement of impulsive attitudes.

For instance, previous studies showed that EDS resulting from accumulated sleep debt was associated with an increased likelihood of exhibiting impulsivity [25,26,27], which, in turn, has been regarded as a predictor of depression in some studies [28,29,30,31]. Hence, it is plausible to hypothesize that impulsivity may mediate the relation between EDS and depression, although there is no general agreement about this mediated effect. Previous studies described that emotional impulsivity may explain the effect of insomnia symptoms on suicide ideation in subjects with bipolar disorders [32], suggesting the potential role of impulsivity as an underlying mechanism linking depressive symptoms to sleep-related difficulties. Therefore, the objective of this study was to take a preliminary step in understanding the relation between EDS and impulsivity in predicting depression among patients with OSAS. A mediation model was hypothesized postulating that impulsivity may mediate this association. Specifically, higher levels of daytime sleepiness may be related to higher levels of impulsivity, which in turn can predict higher depression scores. It was decided to control for the effects of age, sex, and BMI since male sex, high BMI, and older age are undisputable risk factors for OSAS [33]. Accordingly, cumulative evidence showed that the risk of presenting OSAS is higher for males (i.e., having twice the risk), for middle-aged (i.e., 2- to 3-fold higher prevalence) and overweight individuals (i.e., 8- to 10-fold increased risk of OSAS [33,34,35]). Moreover, the AHI as indicator of severity of SDB as well as the oxygen desaturation index (ODI) were also examined as covariates of the proposed model.

Previous findings indicated that daytime sleepiness is a main contributing factor to perceived low quality of life and problematic mental health (e.g., depression) in patients with OSAS, which affects their activities of daily living (e.g., social difficulties, poor job/academic performance) [36]. Therefore, an additional aim was to examine different psychological (e.g., self-esteem, anxiety) and demographic (e.g., smoking behavior, alcohol use) variables among patients with different levels of sleepiness. Furthermore, the trend of these aspects was also examined by stratifying participants according to their OSAS severity, in order to investigate whether psychological difficulties were higher among patients with severe OSAS, as previously indicated [37].

## 2. Materials and Methods

### 2.1. Participants

Subjects consecutively admitted at the Sleep Medicine Clinic of the Neurology Unit of the University Hospital of Rome Tor Vergata were included in this observation. All subjects underwent a sleep medicine visit and completed questionnaires for evaluating their symptoms. Moreover, polygraphic cardiorespiratory monitoring was performed in all subjects for OSAS diagnosis. The diagnosis of OSAS was assigned based on the criteria of the International Classification of Sleep Disorders—3rd Edition. OSAS severity was determined with the AHI index (controls: AHI < 5; mild OSA: AHI ≥ 5 h and <15 h; moderate OSA: AHI ≥ 15 h and <30 h; severe OSA: AHI ≥ 30 h). Moreover, EDS was defined as reflected by an Epworth Sleepiness Scale (ESS) total score ≥ 10 [38].

Exclusion criteria for all subjects included in the study were the following: major psychiatric disorders; neurologic disorders; intake of drugs acting on the CNS; history of alcohol abuse or suicide attempts; shift work.

### 2.2. Instruments

All subjects underwent polygraphic cardiorespiratory monitoring, performed according to AASM criteria [39]. The following parameters were achieved from the polygraphic recording. AHI was defined as the sum of all apneas (>90% reduction in airflow for >10 s) and all hypopneas (>30% reduction in airflow > 10 s) associated with ≥3% O_2_ desaturation [39]. In addition to AHI, the following oxygen saturation (SaO_2_) parameters were measured: mean SaO_2_, lowest SaO_2_, time spent with SaO_2_ < 90% (T < 90), and ODI (number of oxygen desaturations ≥ 3% per hour). Experts in sleep medicine scored the polygraphic recordings (CL, FP, FI).

All the participants completed a sociodemographic form and the following questionnaires:

- ESS [40]. The ESS is a widely used self-report measure to assess subjective daytime somnolence by asking about the likelihood of falling asleep in eight different scenarios (e.g., Sitting and reading, Watching TV). It comprises eight items rated on a four-point Likert scale ranging from 0 (no chance of falling asleep) to 3 (high chance of falling asleep). Higher scores indicate a greater tendency to fall asleep in the relative scenario. This scale demonstrated high reliability in adults with OSA as well as in the general population [41]. The cut-off of 10 is recognized for determining the presence of subjective EDS.
- Barratt Impulsiveness Scale (BIS) [42]. The BIS is one of the most widely instruments to assess trait impulsivity. It comprises a 30-item self-report measure with a four-point Likert scale ranging from Rarely/Never (1) to Almost Always/Always (4). The items assess the following aspects: attentional impulsivity (e.g., “I am restless at lectures or talks”), motor impulsivity (e.g., “I act on impulse”), and non-planning impulsivity (e.g., “I say things without thinking”). Previous findings revealed a good internal consistency for this scale [43].
- Hospital Anxiety and Depression Scale (HADS) [44]. The HADS includes 14 items measuring emotional distress and is divided into 2 sections, respectively assessing depressive (e.g., “Worrying thoughts go through my mind”) and anxiety (“I get sudden feelings of panic”) symptoms. The scale evidenced good reliability and validity for both of these sections [44].
- Rosenberg Self-Esteem Scale (RSES) [45]. The RSS is a measure of self-evaluated global self-esteem consisting of 10 items rated from 1 (“strongly agree) to 4 (“strongly disagree”). One item example is: “I feel that I have a number of good qualities”. A higher score indicates a greater and more reliable high level of individual self-esteem [46].

### 2.3. Statistical Analysis

First, descriptive statistics on demographic, behavioral, and psychological data were observed. After the normality of data was established, group differences were computed considering sleepiness (normal: ESS < 10; EDS: ≥10) and the severity of OSA, as previously stated. More specifically, multivariate analysis of variance (MANOVA) and t-tests were computed in order to examine differences in psychological and demographic variables between patients with different levels of OSA and patients with and without sleepiness, respectively. Furthermore, considering sex differences in OSAS symptom presentation [47] and depression [48], a series of *t-tests* were computed to examine AHI and HADS scores between male and female participants.

Lastly, one mediational model was tested in order to assess whether impulsivity mediated the relationship between sleepiness and depressive symptoms in patients with OSA. The mediation model was tested using Hayes’s PROCESS macro [49] (Model 4) with bootstrapping (5000 resamples of the data) to generate indirect effect estimates with 95% bias-corrected confidence intervals. Confidence intervals that did not include zero indicated significant effects at *p* < 0.05. All beta values (β) reflected standardized coefficients. The indirect effect (αβ) was conceived as the product’s coefficient between sleepiness and impulsivity and the path’s coefficient between impulsivity and depression.

At an older age, being male and obese have been recognized as risk factors for OSA [50,51], so age, sex, and BMI were entered into the regression model as covariates. Moreover, the effects of the AHI and ODI were also tested. Percentage mediation (PM), which is interpreted as the percent of the total effect explained by the indirect effect, was then calculated using the following formula suggested by previous authors: PM = A × B/(A × B + C′) = A × B/C [52]. The analyses were conducted with SPSS 27.0.

## 3. Results

One-hundred and thirty-eight patients (M_age_ = 58.02, SD = 13.83; 60% M) with OSA (M_age_ = 58.84, SD = 13.15; 59% M) and twenty-five controls (M_age_ = 53.65, SD = 13.67; 64% M) admitted to the Sleep Medicine Center of the Neurology Clinic at the University Hospital of Rome “Tor Vergata” were recruited for the present study. Descriptive statistics on behavioral data of the whole sample indicated that 22% and 20% of participants smoked and consumed alcohol, respectively (Table 1).

Results of the multivariate analysis of variance (MANOVA) showed that patients with mild, moderate, and severe OSA did not differ in psychological and demographic variables but differed in BMI. More specifically, post-hoc comparison indicated that patients with severe OSA reported higher BMI (M = 31.61, SD = 5.33) as compared to controls (M = 26.86, SD = 4.87) (F_3,140_ = 5.616; *p* < 0.001).

Results from the t-test assessing group differences according to the severity of sleepiness indicated that participants with EDS reported greater anxiety (t = 2.339; *p* < 0.05), general impulsivity (t = 2.09; *p* < 0.05), attentional impulsivity (t = 3.69; *p* < 0.01) and lower self-esteem scores (t = 3.05; *p* < 0.01) than those without EDS. Results from the t-test assessing sex differences revealed no significant results for either AHI (t = 1.308; *p* = 0.193) or HADS score (t = −0.740; *p* = 0.461).

**Figure 1:**
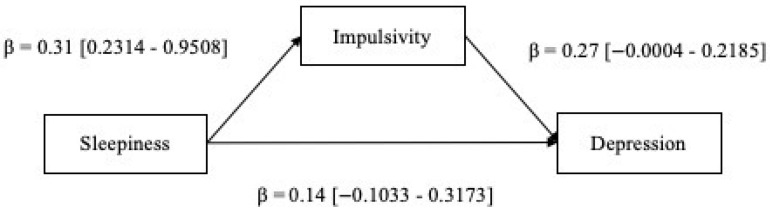
Mediation model. Standardized coefficients are displayed.

Results from the mediation model showed that, while controlling for age, sex, BMI, and OSA severity (i.e., AHI), there was a significant direct effect between sleepiness and impulsivity (β = 0.31, *p* < 0.01) and there was a significant indirect effect of impulsivity in the sleepiness–depression link (αβ = 0.084 [0.0243–0.1617]) (Figure 1). Hence, the hypothesized model was confirmed. The results highlighted the occurrence of a total mediation, as suggested by the non-significance of the path from sleepiness to depression observed when the mediator was entered into the regression. This evidence indicated that the association between sleepiness and depression in patients with OSA is better explained by the mediation of impulsivity. The calculation of the PM index indicated that the effect size of the mediation effect was equal to 37.6%, suggesting the moderate practical significance of the hypothesized model. No statistical significance was observed for the covariates.

## 4. Discussion

OSAS offers a complex clinical presentation generally involving specific psychological alterations and low perceived quality of life [53]. In this study, it was found that participants with different levels of OSA severity did not differ in the main psychological and demographic variables. This evidence concords with previous findings demonstrating no significant associations between OSA severity, sleep problems, and depressive and anxiety symptoms [54,55,56], although it is in contrast with other studies indicating that the OSA severity is associated with higher symptoms of depression and anxiety as well as with higher daytime sleepiness and lower self-esteem [56,57,58]. While it has been observed that depressive and anxiety symptoms are more frequent in individuals with OSAS than in those not affected by this sleep disorder [59], some studies indicated null associations between OSA severity and depression [60,61]. A possible explanation of such inconsistency may be the variability of methodological features of these studies (e.g., study design, patient characteristics, instruments of assessment) [54].

In the present investigation, patients with severe OSA presented a higher BMI as compared to controls, in line with previous studies [38,62]. The association between OSA and BMI has been indicated by both cross-sectional [63] and longitudinal evidence [64], with previous authors documenting a relative risk of OSA from overweight of nearly 10 in overweight individuals [65].

As the literature indicated that psychological difficulties should be suspected in patients with OSAS reporting EDS [57], the present work investigated mean differences in depression, anxiety, impulsivity, and self-esteem scores based on the presence of this disabling and frequent symptom in patients with OSAS. Results indicated that patients with EDS were more prone to report higher anxiety, higher general and attentional impulsivity, and lower self-esteem than those without EDS. These findings reflected previous observations on clinical and psychosocial difficulties of individuals with OSAS and EDS [66]. In particular, previous results indicate a higher frequency of comorbid anxiety in OSA patients with EDS as compared to those without EDS, even after controlling for several confounding factors (e.g., age, sex, BMI) [67]. In terms of general and attentional impulsivity, it has been observed that daytime sleepiness is significantly predicted by higher scores in these domains [26]. Finally, the present results suggest that patients with EDS reported lower self-esteem as compared with the non-EDS group. This finding provided additional support for the significant inverse association between daytime sleepiness and self-esteem found elsewhere [68]. Overall, the results of this study suggest that EDS can be detrimental to a person’s quality of life and emphasize the need to accurately diagnose these conditions, especially considering that OSAS with EDS is frequently unrecognized [36]. Accordingly, EDS represents the more disabling symptoms for patients with OSAS, since it impairs social activities, daily living, well-being, and quality of life [69].

Results of sex differences on OSA severity and depression scores led to non-significant findings. This evidence is somewhat in contrast with previous studies indicating that sex affects the incidence of OSA [70]. More specifically, the literature generally indicates that women present a higher burden of symptoms compared to males, although the AHI can be lower [71]; however, there are also reports showing non-significant sex differences in the AHI [72]. Similarly, female and male patients did not report significant divergent depressive symptoms, inconsistent with the well-known likelihood of females with OSA reporting higher levels of depression than males [73]. Future studies should investigate different clinical manifestations and psychological side effects of OSA in association with sex, in order to examine whether more specific OSA-related aspects (e.g., snoring, sleepiness, negative emotionality) could be more prevalent in males or females.

Results from the mediation analysis revealed that impulsivity fully mediates the association between daytime sleepiness and depression. One previous study [32] has demonstrated that impulsivity can mediate the association between sleep problems and depressive symptoms (e.g., suicide). The present work was focused on EDS, which is a well-known documented symptom of OSAS [57]. It was found that higher levels of daytime sleepiness were associated with higher impulsivity, which, in turn, can predict depressive symptoms. Previous research showed that sleepiness, among the different sleep problems, may lead to exaggerated behavioral reactivity to experiences [26]. Moreover, inadequate sleep is linked to poor impulse control [69], as a reflection of the interaction between sleep deprivation and the immature inhibitory brain circuitry [74]. A possible neurobiological explanation of this mechanism may involve inhibitory brain control.

The results on the association between impulsivity and depression are consistent with previous results [29,30,75,76]. Cumulative evidence indicated impulsivity as a trans-disease process influencing depressive symptoms, although its role in depression may vary depending on the specific facet of impulsivity analyzed [77]. The potential mechanisms underlying the association between impulsivity and depression may involve cognitive distortions such as cognitive rigidity and repetitive negative thinking [78,79], which are cognitive-affective processes characteristic of depressive symptoms [80]. The fact that impulsivity fully mediated the relationship between daytime sleepiness and depression may indicate that this association is not direct, as previous studies asserted [55]; instead, it is better explained by impulsivity. However, cognitive performance was not assessed in subjects included in this study, and this represents a limitation. Future studies should evaluate these associations as well as the proposed mediation model from a longitudinal perspective in order to support causality inferences.

Our results should be interpreted in light of other limitations, including the lack of objective measures of daytime sleepiness. The mere use of self-report questionnaires did not permit the exclusion of social desirability in the responses given by participants but permitted the inclusion of a large population of subjects using a widely used and validated questionnaire, such as ESS. Moreover, notwithstanding the well-known comorbidity between OSA and insomnia (comorbid insomnia and OSA, COMISA, [81]), this aspect was not assessed in our study. Future work should also examine other potential comorbidities of OSA with sleep disturbances. The cross-sectional nature of the study limits any causal interpretation of the mediation model tested. Evidence in the literature substantiated the bi-directional associations between sleepiness and mental distress [30,82] as well as between sleepiness and impulsivity [26]. Therefore, further experimental, possibly longitudinal, investigations are needed to estimate the directionality of these pathways. Some variables that could contribute to depressive symptoms (e.g., psychiatric comorbidities [83], sleep duration [84]) and thus could have confounded the present results were not assessed in this study. An important issue to resolve for future studies is examining how these aspects can be related to depression by examining their covariate role in the proposed mediation model. Finally, the use of impulsivity as a mediator of the sleep–depression relation should be interpreted with caution, considering this trait as a proximal factor of poor impulse control. Future studies should employ other cognitive measures relevant to impulsivity tendencies (e.g., reflection impulsivity, monetary discounting tasks) [85].

Despite the limitations, the present study expanded previous research on the psychological characteristics of patients with OSA and offered compelling evidence of a potential underlying mechanism explaining the association between daytime sleepiness, impulsivity, and depression in these patients. From a practical and clinical perspective, clinicians may pay attention to the implementation of treatment approaches aimed at the reduction of impulsive behaviors and cognition [86] when treating patients with OSA who report depressive symptoms. Furthermore, the present study peripherally suggests that subjective daytime sleepiness represents an important target for intervention strategies since it may reduce impulsivity in patients with OSA, as suggested by experimental studies on the effect of sleep deprivation on increased behavioral disinhibition [87]. Finally, considering the prevalence of residual EDS in patients with OSAS, further studies performed in patients complaining of it are needed. The findings from the mediation model also highlight the role of impulsivity as a promising and intervenable variable, which could be modified through apposite intervention protocols in order to reduce its potential detrimental effect on depressive symptoms. Within this perspective, researchers have documented that mindfulness techniques can attenuate maladaptive impulsivity, since mindfulness and impulsivity are located toward opposite ends of a continuum of awareness and response inhibition [88]. In the light of the present results, it would be advisable for future studies to better refine this promising impulsivity treatment approach by also exploring the role of sleep patterns in individuals’ psychological distress targeted for intervention. In conclusion, this study highlighted the importance of evaluating the wide spectrum of psychological symptoms in patients with OSA, considering the interconnections among daytime sleepiness, depression, and impulsivity and the importance of not overlooking these symptoms to ensure treatment compliance and patients’ well-being.

## Figures and Tables

**Table 1:** Demographic characteristics.

	Total Sample *n* (%)	M ± SD	Range	OSA *n* = 138	Controls *n* = 25
Age		58.02 ± 13.83	16–83	58.84 ± 13.15	53.65 ± 13.67
Sex	98 M (60%)		-	*n* = 82 M (59%)	*n* = 16 M (64%)
Education (years)		11.90 ± 4.41	5–28	11.99 ± 4.53	11.45 ± 3.71
BMI		29.31 ± 5.41	17.33–44.63	29.74 ± 5.40	26.86 ± 4.87
(Response: yes/no)					
Smokers	36 (22.8%)		-	*n* = 31 (23%)	*n* = 5 (20.8%)
Number of cigarettes a day		11.65 ± 7.12	1–25	11.93 ± 7.33	10 ± 6.12
Alcohol consumption	34	-	-	*n* = 28 (20.9%)	*n* = 6 (25%)
Physical activity	55 (35%)	-	-	*n*= 48 (36.1%)	*n* = 7 (29.2%)
Enough bodily movement	52 (33.8%)	-	-	*n* = 43 (33.1%)	*n* = 9 (37.5%)
Dinner wine	30 (19%)	-	-	*n* = 23 (17.3%)	*n* = 7 (29.2%)

## Data Availability

The data presented in this study are available on request from the corresponding author.
